# Case Studies and Literature Review of *Francisella tularensis*–Related Prosthetic Joint Infection 

**DOI:** 10.3201/eid2906.221395

**Published:** 2023-06

**Authors:** Léa Ponderand, Thomas Guimard, Estibaliz Lazaro, Henry Dupuy, Olivia Peuchant, Nathalie Roch, Philippe Deroche, Tristan Ferry, Max Maurin, Aurélie Hennebique, Sandrine Boisset, Isabelle Pelloux, Yvan Caspar

**Affiliations:** Grenoble Alpes University Hospital Center, Grenoble, France (L. Ponderand, M. Maurin, A. Hennebique, S. Boisset, I. Pelloux, Y. Caspar);; Univ. Grenoble Alpes, CNRS, CEA, IBS, Grenoble, France (L. Ponderand, S. Boisset, Y. Caspar);; Departmental Hospital Center of Vendée, La Roche sur Yon, France (T. Guimard);; University Hospital Center of Haut-Lévêque, Pessac, France (E. Lazaro, H. Dupuy);; University Hospital Center of Bordeaux, Bordeaux, France (O. Peuchant);; William Morey Hospital Center, Chalon-sur-Saône, France (N. Roch);; Dracy-le-Fort Orthopedic Center, Dracy-le-Fort, France (P. Deroche);; Claude Bernard University Lyon 1, CNRS ENS Lyon, INSERM, Lyon, France (T. Ferry);; Croix-Rousse University Hospital Center, Hospices Civils de Lyon, Lyon (T. Ferry);; Univ. Grenoble Alpes, CNRS, Grenoble INP, TIMC-IMAG, Grenoble (M. Maurin, A. Hennebique)

**Keywords:** tularemia, prosthetic joint infection, *Francisella tularensis*, zoonotic disease, zoonoses, bacteria, France

## Abstract

Tularemia is a zoonotic infection caused by *Francisella tularensis*. Its most typical manifestations in humans are ulceroglandular and glandular; infections in prosthetic joints are rare. We report 3 cases of *F. tularensis* subspecies *holarctica*–related prosthetic joint infection that occurred in France during 2016–2019. We also reviewed relevant literature and found only 5 other cases of *Francisella*-related prosthetic joint infections worldwide, which we summarized. Among those 8 patients, clinical symptoms appeared 7 days to 19 years after the joint placement and were nonspecific to tularemia. Although positive cultures are typically obtained in only 10% of tularemia cases, strains grew in all 8 of the patients. *F. tularensis* was initially identified in 2 patients by matrix-assisted laser desorption/ionization time-of-flight mass spectrometry; molecular methods were used for 6 patients. Surgical treatment in conjunction with long-term antimicrobial treatment resulted in favorable outcomes; no relapses were seen after 6 months of follow-up.

*Francisella tularensis* is a fastidious, gram-negative coccobacillus that can cause tularemia, a zoonotic disease. Two subspecies are responsible for human cases: *F. tularensis* subspecies *tularensis* (type A strains) and *F. tularensis* subsp. *holarctica* (type B strains) (*1*). Tularemia is a reemerging disease that has occurred recently both sporadically and in outbreaks worldwide. No vaccines are available, and antibiotic classes effective in treatment are limited to aminoglycosides, fluoroquinolones, and tetracyclines (*2*,*3*). 

Potential reservoirs and vectors in both the terrestrial and aquatic cycles of this bacterium are varied. Six main clinical forms of tularemia have been described (glandular, ulceroglandular, oropharyngeal, oculoglandular, pneumonic, and typhoidal), depending on the route of bacterial inoculation. Tularemia can be transmitted through direct contact with infected animals (hares, rabbits, small rodents, etc.); through the bites of blood-sucking arthropods; through consumption of contaminated food or water; through conjunctival inoculation with contaminated fingers, materials, or aerosolized particles; or through the lungs, either by inhaling infectious aerosols or by the hematogenous spread of bacteria (*4*–*7*). 

Severe infections are predominantly associated with *F. tularensis* subsp. *tularensis*, which is present only in North America, whereas *F. tularensis* subsp. *holarctica*, the only subspecies found in both Europe and North America, largely causes incapacitating and chronic disease with large or multiple lymphadenopathies (*8*). According to data from the French National Reference Center for *Francisella* (FNRCF) and from mandatory notifications to the French Public Health Agency, the ulceroglandular and glandular forms account for most (72%) clinical forms of the disease (*9*). Bone and joint infections (BJIs) and prosthetic joint infections (PJIs) related to *F. tularensis* are extremely rare and have been reported sporadically in literature (*10*–*13*). BJIs are primarily related to staphylococci, streptococci, or gram-negative rods, but any bacterial species can cause an infection in the presence of prosthetic material (*14*,*15*). We report 3 cases of *F. tularensis*–related PJIs occurring during 2016–2019 in France, as well as the results of a literature review on BJIs and PJIs related to this highly pathogenic bacterium (Tables 1, 2).

**Table 1:** Clinical and biologic characteristics of patients with *Francisella tularensis*–related prosthetic joint infection*

Characteristic	Case							
	1	2	3	4 (*10*)	5 (*11*)	6 (*11*)	7 (*12*)	8 (*13*)
Country	France	France	France	United States	Switzerland	Czech Republic	United States	Canada
Region	Vendée	Gironde	Aube	Colorado	NS	NS	Illinois	Ontario
Age, y	49	70	68	58	84	84	77	68
Sex	M	M	M	M	F	M	M	M
Exposure factors	Rural residence, dog	Hunter	Retired butcher, cooking with wild game	Farmer, possible rabbit carcass handling	Airborne transmission (rabbit barn in neighbor’s house)	Walk in tularemia- endemic area	Hunter	Hunter, tick bite 6 mo before surgery
Type of surgery	Left THR	Left TKA	Left THR	Left TKA	Right knee prosthesis	Right TKA	Right THR	Right TKA
Delay between diagnosis and prosthesis implantation	35 d	12 y	19 y	9 mo	12 y	8 y	7 d	6 mo
Clinical symptoms	Infected scar, fever	Feverish confusion, bilateral mediastinal and hilar lymphadenopathy, knee swelling	Joint pain	Repeated joint effusion	Erythema, joint pain	Fever, abdominal pain, confusion, painful knee effusion	Fever, joint pain and swelling, bullous skin lesion with itching	Joint pain, warm and swollen knee
CRP, mg/L	21	100	58	NA	81	98	16	NA
Leukocytes, G/L	4.45	4	NA	NA	NA	NA	NA	NA
Sample type	Abscess	Joint aspiration	3 tissues	Joint aspiration	7 tissues	Joint aspiration	Joint aspiration	Joint aspiration
No. positive samples/total	1/1	1/1	2/3	1/1	6/7	1/1	1/1	1/1
Delay to positivity	NA	7 d	5 d	24 h–48 h	12 d	4 d	7 d	3 d
Identification	Vitek 2 GN Biochemical assay; ISFtu2 PCR +; specific *F. tularensis* subspecies*holarctica* PCR	Specific *Francisella* PCR; ISFtu2 PCR +; 16S-23S PCR + sequencing	MALDI-TOF MS (Microflex LT); ISFtu2 PCR +; specific *F. tularensis* subspecies *holarctica* PCR	Sequencing	16S rRNA gene sequencing	16 S rRNA gene sequencing	MALDI-TOF MS	Sequencing
Serologic results	MAT 640; IFA IgG 1,280; IgM 640	ELISA IgG 1.5; IgM 3.58; IFA IgG 640; IgM 640	MAT 160	ND	IgM 232.6 U/mL; IgG 126.4 U/mL	MAT 80	Positive (titers NA)	MAT 320

*CRP, C-reactive protein; IFA, immunofluorescence assay; MALDI-TOF MS, matrix-assisted laser desorption/ionization time-of-flight mass spectrometry; MAT, microagglutination test; Microflex LT, Bruker Daltonics; NA, not available; ND, not done; NS, not specified; THR, total hip replacement; TKA, total knee arthroplasty; +, positive.

**Table 2:** Surgery, antibiotic treatment, and follow-up of patients with *Francisella tularensis*-related prosthesis joint infection

Characteristic	Case							
	1	2	3	4 (*10*)	5 (*11*)	6 (*11*)	7 (*12*)	8 (*13*)
Surgery	1-stage revision joint replacement	1-stage revision joint replacement	1-stage revision with partial joint replacement	Repeated joint aspiration	2-stage revision joint replacement	Repeated joint aspiration	DAIR	2-stage revision joint replacement
Antibiotic treatment								
Before surgery	DOX 100 mg 2×/d until surgery	OFX 200 mg 2×/d for 6 wk	ND	ND	AMC	AMC	ND	IV cloxacillin 2 g/6 h for 10 d, oral cloxacillin 500 mg 4×/d
After surgery	CIP 750 mg 2×/d + DOX 100 mg x2 2×/d for 9 wk	IV CIP 500 mg 2×/d + IV AMK 1,200 mg for 5 d, CIP 500 mg 2×/d for 6 wk	CIP 750 mg 2×/d 3 mo, lifetime treatment by DOX 100 mg 2×/d	DOX, dosage NA	DOX 100 mg 2×/d for 6 wk	DOX 100 mg 2×/d for 20 d + GEN 240 mg for 10 d, CIP 500 mg 2×/d for 20 d	DOX 100 mg 2×/d for 12 mo	IV CFZ 2g/8h 6 wk + RIF 300 mg 2×/d, CIP 500 mg 2×/d + RIF 300 mg 2×/d >6 mo
Progress at follow up	No relapse, no biologic inflammatory limp, chronic joint pain	Biologic surveillance, favorable evolution	Favorable evolution with mild limp	No relapse, persistence of knee swelling	Favorable evolution	Small pain-free effusion at 24 mo	Resolution of pain and skin lesion	Resolution of symptoms under treatment
Staff monitoring or prophylaxis	Prophylactic DOX or CIP	NA	Clinical and serologic	NA	Serologic	Prophylactic DOX	NA	NA

*AMC, amoxicillin/clavulanic acid; AMK, amikacin; CFZ, cefazolin; CIP, ciprofloxacin; DOX, doxycycline; DAIR, debridement, antibiotics, implant retention; GEN, gentamicin; IV, intravenous; NA, not available; ND, not done; OFX, ofloxacin; RIF, rifampin.

## Material and Methods

The following methods and laboratory precautions were used at the FNRCF to confirm the diagnosis of tularemia. For *F. tularensis* culturing, samples and strains were housed in a Biosafety Level 3 laboratory. Samples were seeded on chocolate agar and incubated at 37°C in a CO_2_-enriched atmosphere for 10 days. Methods for identifying *F. tularensis* subsp. *holarctica* varied among the 3 patient cases described in this article. Before 2018 (case 1), specific real-time insertion sequence *F. tularensis* (ISFtu2) PCR and sequencing of the 16S 23S intergenic region (*16*) were used to confirm identification at the subspecies level. Since 2018 (cases 2 and 3), *F. tularensis* subsp. *holarctica* identification has been confirmed using in-house ISFtu2 PCR and *F. tularensis* subsp. *holarctica*–specific PCR, according to previously published protocols (*17*,*18*). Before 2018 (case 1), serologic testing was performed using in-house microagglutination test (MAT) and in-house indirect immunofluorescence assay (IFA). Since 2018, ELISA (Serion Diagnostics) (case 2) and chemiluminescent immunoassay (Vircell) have replaced the MAT method. 

For this study, we performed retrospective antibiotic susceptibility testing to determine the MIC of 7 antibiotics (gentamicin, ciprofloxacin, levofloxacin, rifampin, erythromycin, azithromycin, and doxycycline). We used an in-house broth microdilution method, as previously described (*19*).

For the retrospective case search, we performed a PubMed search for published cases of PJI caused by *F. tularensis* using the terms “*Francisella* AND joint AND infection” with no restriction of date or language. In total, 21 articles matched the searched criteria, but only 5 cases corresponded to PJI.

## Results

### Case 1

**Figure 1:**
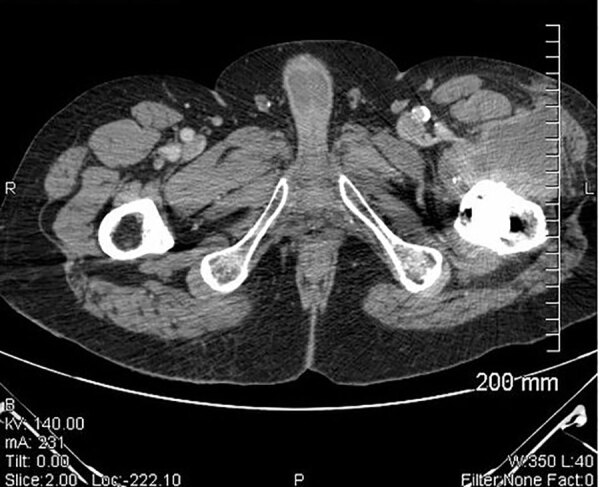
Computed tomography scan of pelvis in case 1 showing large abscess of the left hip with a periosteal image compatible with an osteitis in case of *Francisella tularensis*–related prosthetic joint infection, France.

A 49-year-old man living in Vendée County, France, was hospitalized in March 2016 for a left femoral neck fracture after a fall. The patient underwent hip arthroplasty with prosthesis placement. His medical history included active smoking and alcoholic cirrhosis since 2008. One month later, the patient was transferred to the intensive care unit for hepatic encephalopathy. During his hospitalization, an abscess developed near the prosthesis scar; surgical site infection was suspected. A computed tomography (CT) scan of the pelvis revealed soft tissue infection of the left hip; the periosteal image was compatible with osteitis (Figure 1). The patient’s blood culture results were negative, C-reactive protein (CRP) was 21 mg/L (reference <10 mg/L), and leukocyte count was 4.45 giga (G)/L (4.1–9.9 G/L). After 2 days, a specimen from the abscess demonstrated growth of a small gram-negative coccobacillus on chocolate agar under 5% CO_2_ enrichment, which was not identified by VitekMS matrix-assisted laser desorption/ionization time-of-flight (MALDI-TOF) mass spectrometry (bioMérieux). *F. tularensis* was suspected on the basis of results of the Vitek 2 GN biochemical assay (bioMérieux) 1 day later. The strain was sent to the FNRCF, where *F. tularensis* subsp. *holarctica* infection was confirmed. Serologic results from MAT were positive (640 [reference <80]), and elevated IgM (640 [reference <80) and IgG (1,280 [reference <80]) titers were confirmed by IFA. The patient received a course of doxycycline (100 mg 2×/d for 1 mo), then underwent a 1-stage revision joint replacement; respiratory precautions (KN95 masks) were in place for all persons in the operating theater to prevent secondary transmission through contaminated droplets. Gentamicin (3 mg/kg) was administered during surgery, followed by ciprofloxacin (750 mg) and doxycycline (100 mg 2×/d), both for 9 weeks. Five samples collected during surgery remained sterile. 

Although the infection was cured, the patient still had joint pain without fever or biologic inflammatory syndrome 4 years later. The patient was not a hunter but lived in a rural, *Francisella*-endemic area of France and owned a dog, which sometimes brought wild animals or game that could have been infected. The patient did not eat game meat, however, and he did not leave the hospital between the initial prosthetic hip surgery and the appearance of the abscess. We proposed 2 hypotheses to explain the infection: contamination occurred a few days before the patient’s fall and bacteria were already present in his blood, leading to prosthesis contamination and abscess; or contamination occurred intraoperatively because of skin contamination after the fall. Multiple skin scratches, bruises, and wounds were described. Unfortunately, no serum samples were available to assess the date of contamination. Monitoring of laboratory staff included prophylactic treatment with doxycycline or ciprofloxacin. Because surgeons were protected with gloves, glasses, and surgical masks, and no injuries were reported involving contaminated materials, no monitoring was performed.

### Case 2

**Figure 2:**
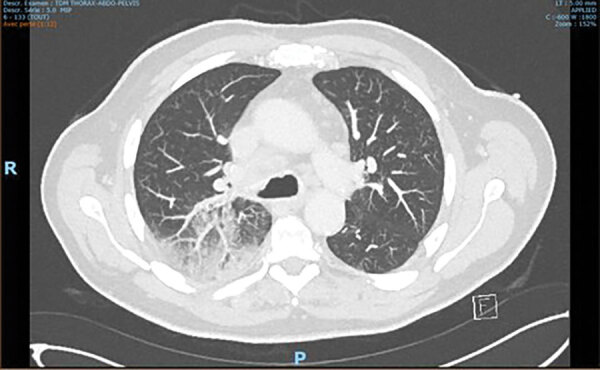
PET scan of the lung in case 2 showing the presence of bilateral mediastinohilar lymphadenopathies associated with a diffuse condensation of the upper right pulmonary lobe in case of *Francisella tularensis*–related prosthetic joint infection, France.

A 70-year-old man was hospitalized in August 2018 with fever, confusion, headache, cervical pain, and lower limb weakness. He lived in a forested rural area in Gironde County, France, and had been experiencing asthenia and anorexia for several months. His medical history included diabetes, high blood pressure, dyslipidemia, and regular alcohol consumption. He underwent a left knee replacement with total knee prosthesis in 2006. Laboratory examinations revealed a CRP level of 100 mg/L (reference <10 mg/L) and leukocyte count of 4 G/L (4.1–9.9 G/L). Cerebrospinal fluid (CSF) and blood culture samples were taken, and treatment with ceftriaxone and acyclovir was initiated. The culture of both samples remained sterile, and results of herpes simplex virus/varicella zoster virus PCR were also negative. The patient had not traveled abroad but had birds at home (hens, pigeons, and doves) and was a fisherman and hunter. CT and positron emission tomography demonstrated bilateral mediastinal and hilar lymphadenopathies associated with diffuse condensation of the upper right pulmonary lobe, compatible with infection (Figure 2). *Legionella pneumophila* and *Streptococcus pneumonia* urinary antigen test results were negative. Bronchoalveolar lavage did not identify any pathogen, but white blood cell count identified 60% gamma-delta T cells in the lymphocyte fraction (40% of total leukocytes), possibly indicating infection with an intracellular bacterium. At a follow-up visit 20 days later, the patient felt better but was still experiencing evening fever (38°C) without chills or night sweats. Clinical examination revealed swelling of the left knee. Joint aspiration revealed a leukocyte count of 99,000 cells/mm^3^ (reference <1,700 cells/mm^3^) with 70% lymphocytes. Unexpectedly, gray-white, smooth, and small colonies grew after 7 days on chocolate agar under 5% CO_2_ enrichment; the bacteria were identified as *F. tularensis* by PCR targeting the gene encoding *F. tularensis* outer membrane protein A after failure to confirm identification with Biotyper MALDI-TOF mass spectrometry (Bruker Daltonics). Results of a *Francisella*-specific PCR performed directly on joint aspirate was also positive. The strains and samples were sent to FNRCF, where the strain was confirmed as *F. tularensis* subsp. *holarctica*. Results of the mediastinal lymph node biopsy, however, were PCR-negative. 

Serologic samples from early September 2018 showed elevated IgM titers of 3.58 (reference <0.45) and IgG titers of 1.5 (reference <0.62) by ELISA (Serion Diagnostics) and titers of 640 (IgM) and 640 (IgG) by IFA, corroborating recent *F. tularensis* infection. Antibiotic therapy with ofloxacin (200 mg 2×/d) was administered for 6 weeks, and a 1-stage joint replacement was performed in March 2019. Of 5 samples taken during surgery, 2 were found to be positive for *Francisella* by ISFtu2 PCR; however, because of a small bacterial concentration, results for the specific but less sensitive *F. tularensis* subsp. *holarctica* PCR remained negative. The fifth culture was sterile. Ciprofloxacin (500 mg 2×/d) and amikacin (1,200 mg 1×/d) were started during surgery; amikacin was stopped after 5 days. By April, the wound was sufficiently healed and CRP had decreased to 13.5 mg/L, enabling the patient to return home; w ciprofloxacin was continued for 6 weeks. His progress was favorable for clinical and biologic healing. 

The suspected mode of contamination was infection by animal or aerosol, and initial pulmonary symptoms indicated likely airborne contamination that could have occurred by inhaling contaminated aerosols or dust. The patient was fishing at the time symptoms appeared, so inhaling contaminated water droplets seems a plausible source because of the possible aquatic reservoir of this bacterium (*4*,*20*).

### Case 3

A 68-year-old man living in Aube County, France, with a medical history of left hip prosthesis implantation in 2000 and right knee prosthesis implantation in 2008 was evaluated in August 2019 for lateral discomfort on his left side, functional hip difficulties, and pain adjacent to his hip prosthesis. Radiography revealed no major prosthesis degradation. Scintigraphy was positive, however, and CT performed in September revealed wear and a cavity in the metal socket of the prosthesis. A 1-stage revision of the joint was performed in October 2019 to replace the metal socket of the prosthesis. The stem was not unsealed and was therefore left in place; the surgeon noted a joint exudate and fibrosis during the operation. CRP was 58 mg/L. Unexpectedly, 2 of 3 samples obtained during surgery grew a fastidious Gram-negative coccobacillus, identified as *F. tularensis* by Biotyper MALDI-TOF mass spectrometry after 5 days on chocolate agar of aerobic cultures with 5% CO_2_ enrichment. The strains and samples were sent to FNRCF, where the strain was confirmed as *F. tularensis* subsp. *holarctica*. Results of MAT (160 [reference <20]) were positive. Ciprofloxacin (750 mg 2×/d) was administered for 3 months. 

After requesting a notice from Centre de Référence des Infections Ostéo-Articulaires Complexes, the reference center for the management of BJIs, and because the stem of the initial prosthesis was in place, lifetime antibiotic treatment with doxycycline (100 mg 2×/d) was recommended; long-term oral suppressive antibiotic therapy to maintain a functioning prosthesis can be recommended when removing all the components of the prosthesis is not possible. The patient’s progress was favorable; he demonstrated good healing and biologic inflammatory syndrome regression and had a slight limp after 3 months. The patient was a retired butcher who often made pâté with wild game, which probably led to contamination by direct contact with infected animals or meat. No monitoring was performed for surgeons and surgical staff. However, laboratory staff follow-up care included clinical and serologic monitoring.

### Laboratory Safety

For all 3 cases, before *F. tularensis* was suspected or confirmed, samples and strains were handled under Biosafety Level 2 conditions in clinical microbiology laboratories. Samples were rapidly sent to the FNRCF to confirm identification. MIC determinations were performed a posteriori on the 3 strains from France (Table 3). MICs were the lowest for ciprofloxacin (0.016–0.032 mg/L) and levofloxacin (0.032 mg/L), and no resistance to aminoglycosides, rifampin, fluoroquinolones, or tetracyclines was observed. The erythromycin MIC of 2 mg/L confirmed that the 3 strains belonged to biovar I of *F. tularensis* subsp. *holarctica* (*19*,*21*).

**Table 3:** MICs of 3 strains of *Francisella tularensis* subspecies *holarctica* obtained by broth microdilution method, France*

Antibiotics	MICs, mg/L			Breakpoints for susceptibility, mg/L
	Case 1	Case 2	Case 3	
Gentamicin	0.5	0.5	0.5	4†
Ciprofloxacin	0.016	0.032	0.016	0.25†
Levofloxacin	0.032	0.032	0.032	0.5†
Rifampin	0.5	0.5	0.5	1‡
Erythromycin	2	2	2	16‡
Azythromycin	1	1	1	4‡
Doxycycline	0.25	0.25	0.25	4†

*The assay medium was CAMHB supplemented with 2% PolyViteX (CAMHB-PVX; bioMérieux) and adjusted to pH 7.1+0.1 as recommended (17). MICs were read after 48 h of incubation and were interpreted using the Clinical and Laboratory Standards Institute susceptibility breakpoints for *Francisella tularensis* when available or the *Haemophilus influenzae* European Committee on Antimicrobial Susceptibility Testing breakpoints. †Clinical and Laboratory Standards Institute breakpoint. ‡*H. influenzae* European Committee on Antimicrobial Susceptibility Testing breakpoint.

## Discussion

Prosthetic joint replacement is a common surgery increasingly used to improve quality of life (*22*,*23*). Several recommendations to limit infection at the surgical site exist (*24*–*26*), which are either preoperative (skin disinfection by shower with antiseptic or antimicrobial soap, nasal and cutaneous decolonization of *S. aureus*), perioperative (perioperative antiseptic use, intraoperative systemic antimicrobial prophylaxis, asepsis of the operative environment), or postoperative (postoperative antimicrobial prophylaxis). Despite those preventive measures, prosthetic joint infections occur in 1%–2% of patients (*27*–*29*). The most common pathogens described are staphylococci (*S. aureus* and coagulase-negative staphylococci), isolated in 50%–60% of the cases; streptococci; and enterococci (together accounting for 10%). Aerobic gram-negative rods and anaerobic bacteria are isolated from <10% of knee and hip prosthetic joint infections. However, 5%–34% of prosthetic infections remain culture-negative because of previous antimicrobial treatment or because fastidious or low-inoculum pathogens can remain undetectable by classic culture or PCR methods (*27*,*28*,*30*).

Prosthetic joint infection is an extremely rare form of tularemia; we found only 5 cases reported in the literature to date (Tables 1, 2) (*10*–*13*). Of the 8 cases of prosthetic infections summarized in this article, 7 (88%) occurred in men >49 years of age (range 49–84 years). The knee prosthesis was infected in 5 (62.5%) patients and hip prostheses in 3 (37.5%) patients. In all but 2 patients, clinical symptoms appeared >6 months after joint placement (7 days–19 years, median 4 years) and were unspecific to tularemia. Fever, joint pain, and joint exudate were often described, and inflammatory blood markers, where reported, usually increased (CRP range 16–100 mg/L). Inoculation of *F. tularensis* at the surface of the prosthesis seems to be possible in 3 different ways: through direct transfer from contaminated skin wounds or ulcers during surgery (case 1), after surgery through direct skin contamination close to the surgical site scar, or by hematologic spread of the bacteria from an initial infection site distant from the prosthesis (cases 2 and 3). In the reported BJI cases, the initial contamination was suspected to occur through direct transmission from an animal reservoir, either by ingestion of undercooked meat prepared from an infected animal or contact with infected carcasses (cases 2, 3, 4, 7, and 8); possibly by an arthropod bite, as in case 8, in which the patient noted a tick bite 6 months before his initial knee arthroplasty; or by inhalation of contaminated aerosols or dust (cases 1, 5, and 6). For some patients, infectious symptoms were absent or mild, suggesting that the bacteria might be able to survive in a quiescent form with low virulence levels after seeding on the prosthesis. Mechanisms similar to those involved in the long-term persistence of *F. tularensis* subsp. *holarctica* in soil and water might occur, such as switching to a viable but noncultivable state or biofilm formation (*4*; C.D. Brunet et al., unpub. data, https://www.biorxiv.org/content/10.1101/2022.02.18.480867v3). Biofilm formation has been described for the *Francisella* species in vitro and might be a key mechanism in human pathogenesis and infection, especially PJIs (*28*,*31*).

According to the World Health Organization definition of tularemia, presumptive cases are defined as cases in persons with clinical symptoms compatible with tularemia and either positive DNA detection in 1 clinical sample or a single positive serum sample. An infection is confirmed when an *F. tularensis* strain is isolated and identified in culture or by a 4-fold increase in IgM or IgG titers in paired serum specimens (*17*). Although tularemia is primarily identified by serologic assays, the proportion of diagnoses assessed by molecular methods on tissue samples is increasing because of the development of specific PCRs or the availability of 16S rDNA sequencing. Positive bacterial cultures are usually obtained in <10% of cases (*7*,*16*) because *F. tularensis* is a fastidious bacterium that requires cysteine-supplemented agar (*17*). Growth occurs in 2–4 days of incubation at 37°C in aerobic or CO_2_-enriched atmosphere, and colonies are gray-white, round, and smooth with a small halo (*32*,*33*). Surprisingly, in these 8 cases, the diagnosis was made by positive culture on joint aspiration or perioperative tissues. This finding highlights the major need for rapid seeding of fresh tissues on an agar media to enable the growth of this fastidious bacterium from human samples (*34*). Serologic testing, when performed, identified mostly high titers in MAT, ELISA, or IFA, confirming *Francisella* infection, but was performed after the initial diagnosis. The identification of 1 strain was suspected on the basis of results of the Vitek 2 GN assay. MALDI-TOF mass spectrometry enabled identification of 2 strains but did not provide subspecies distinction (*35*–*37*). *F. tularensis* has been identified using the in vitro diagnosis database of the Biotyper mass spectrometer since 2017 (partial integration of 5 species of the security-relevant library as a library extension) only if the extension was added to the database by the user. In contrast, the Vitek MS database does not contain any *F. tularensis* spectra. In 7 of 8 cases, the strain was identified or confirmed by molecular methods (Table 1). Because of the high level of 16S rDNA similarity among the *Francisella* species (98.5%–99.9%), 16S rDNA amplification and sequencing enables identifying the bacterium only to the genus level (*38*). Species or subspecies determination requires PCR targeting specific genes, such as *ISFtu2*, *23kDa*, *tul4*, or *fopA* (*39*). Some of those targets, however, might cross-react with *F. novicida*, *F. philomiragia*, or *Francisella*-like endosymbionts. Additional techniques are necessary to identify *F. tularensis* subspecies, such as PCR targeting a junction between ISFtu2 and a flanking 3′ region (*18*), identification on the basis of size differences of amplified DNA products (Ft-M19, ISFtu2, RD1, and pdpD-2 assay) (*17*,*40*), and amplification and sequencing of the 16S-23S rRNA intergenic spacer region (*16*).

Strategies for PJI treatment combine surgical interventions and antimicrobial therapy. Overall, in cases of early infection (<15 days after prosthesis placement), prosthesis retention with debridement is recommended. In chronic infection, prosthesis removal is the best option, performed as either a 1-stage or 2-stage replacement procedure, depending on the patient history, the bacterium identified, and susceptibility to antibiotics. Those surgical treatments are associated with a long duration (4–6 weeks) of antimicrobial therapy adapted to the antibiotic susceptibility of the bacteria identified (*28*,*41*,*42*). Several guidelines for tularemia treatment have been published (*2*,*17*,*43*,*44*). The antibiotic classes recommended for first-line treatment of tularemia are aminoglycosides, fluoroquinolones, and tetracycline because the bacterium is intrinsically resistant to many other antibiotic classes (all β-lactams, TMP/SXT, clindamycin, glycopeptides, and daptomycin) (*19*). For severe tularemia cases, parenteral gentamicin (5 mg/kg/d) treatment is recommended depending on the clinical response. In moderate cases, oral ciprofloxacin (800–1,000 mg/d) or doxycycline (200 mg/d) can be administered for a minimum duration of 10–14 days. However, those recommendations have not yet been established for BJIs. Of the 8 cases we reviewed, 5 patients underwent 1- or 2-stage revision joint replacement; 1 underwent debridement, antibiotic, and implant retention; and 2 underwent regular joint aspiration without prosthesis replacement. For 2 patients, aminoglycosides were administered during and after the surgery (120 mg amikacin for 5 days and 240 mg gentamicin for 10 days), followed by monotherapy with ciprofloxacin (500 mg 2×/d for 20 days). Three patients received monotherapy; 1 patient took ciprofloxacin (750 mg 2×/d) for 3 months, and 2 patients were prescribed doxycycline (100 mg 2×/d) for 6 weeks or 12 months. Combination therapy was administered in 2 other cases, consisting of ciprofloxacin with rifampin for >6 months or ciprofloxacin with doxycycline for 9 weeks. When information was available, the patients’ follow-up visits revealed favorable progress without joint infection relapse.

Strains have not been reported that are resistant to the recommended first-line antibiotic classes. A comprehensive review reported low MICs for ciprofloxacin (<0.002–0.125 mg/L), gentamicin (<0.016–2 mg/L), and doxycycline (0.064–4 mg/L) against *F. tularensis* strains (*19*). Aminoglycosides penetrate slowly intracellularly and are effective against extracellular bacteria. On the basis of our previous comprehensive review of antimicrobial susceptibility testing data in vitro, in cellular model, and in mice model of infection (*19*), we advise using aminoglycoside for only a short period during and after surgery, when a bacteremic phase can occur, to rapidly kill extracellular bacteria and prevent hematologic spread after surgery. Combining aminoglycosides with ciprofloxacin might confer the highest efficacy because of the rapid penetration of fluoroquinolones in bones and joint tissues and their efficient activity against the intracellular niche of *Francisella*. Thus, combining aminoglycosides and fluoroquinolones might be considered in these severe cases (*44*). Ciprofloxacin and doxycycline, alone or in combination, can be used for long-term treatment. Interest in combination therapy with rifampin has not been demonstrated, but in vitro activity of rifampin against *Francisella* has been observed (MICs range 0.094–3 mg/L), and its excellent bone diffusion might enhance successful outcomes, as demonstrated by case 1 (*13*,*19*,*21*,*45*). However, rifampin is not recommended for tularemia treatment because of insufficient in vivo data (*19*).

Because of the difficulty and delay involved in identifying *Francisella* strains, laboratory staff can be exposed to bacteria without recommended precautions. During surgical interventions, surgeons can be exposed through contact with infectious materials, accidental inoculation, or exposure to aerosols and infectious droplets. World Health Organization guidelines distinguish 3 situations: proven accidental laboratory exposure, potential exposure to *F. tularensis* aerosols, and unlikely exposure. Antibiotic prophylaxis (1,000 mg ciprofloxacin or 200 mg doxycycline for 14 days) or clinical follow-up was recommended according to exposure risk (*17*).

In conclusion, PJI is an unusual clinical manifestation of tularemia that might be underestimated because of the fastidious culture conditions and difficulty in strain identification. Infection might occur in tularemia-endemic areas or in the presence of risk factors. The combination of surgical and extended antibiotic treatment generally leads to favorable outcomes.

## References

[1] Oyston PCF, Sjostedt A, Titball RW. Tularaemia: bioterrorism defence renews interest in *Francisella tularensis.* Nat Rev Microbiol. 2004;2:967–78. 10.1038/nrmicro1045 15550942

[2] Boisset S, Caspar Y, Sutera V, Maurin M. New therapeutic approaches for treatment of tularaemia: a review. Front Cell Infect Microbiol. 2014;4:40. 10.3389/fcimb.2014.00040 24734221 PMC3975101

[3] Maurin M, Mersali NF, Raoult D. Bactericidal activities of antibiotics against intracellular *Francisella tularensis.* Antimicrob Agents Chemother. 2000;44:3428–31. 10.1128/AAC.44.12.3428-3431.2000 11083651 PMC90216

[4] Hennebique A, Boisset S, Maurin M. Tularemia as a waterborne disease: a review. Emerg Microbes Infect. 2019;8:1027–42. 10.1080/22221751.2019.1638734 31287787 PMC6691783

[5] Burckhardt F, Hoffmann D, Jahn K, Heuner K, Jacob D, Vogt M, et al. Oropharyngeal tularemia from freshly pressed grape must. N Engl J Med. 2018;379:197–9. 10.1056/NEJMc1800353 29996079

[6] Eren Gok S, Kocagul Celikbas A, Baykam N, Atay Buyukdemirci A, Eroglu MN, Evren Kemer O, et al. Evaluation of tularemia cases focusing on the oculoglandular form. J Infect Dev Ctries. 2014;8:1277–84. 10.3855/jidc.3996 25313604

[7] Maurin M, Gyuranecz M. Tularaemia: clinical aspects in Europe. Lancet Infect Dis. 2016;16:113–24. 10.1016/S1473-3099(15)00355-2 26738841

[8] Petersen JM, Molins CR. Subpopulations of *Francisella tularensis ssp. tularensis* and *holarctica*: identification and associated epidemiology. Future Microbiol. 2010;5:649–61. 10.2217/fmb.10.17 20353304

[9] Mailles A, Vaillant V. 10 years of surveillance of human tularaemia in France. Euro Surveill. 2014;19:20956. 10.2807/1560-7917.ES2014.19.45.20956 25411688

[10] Azua EN, Voss LA. Tularemia in a prosthetic joint infection. Orthopedics. 2020;43:e54–6. 10.3928/01477447-20190627-01 31269216

[11] Chrdle A, Trnka T, Musil D, Fucentese SF, Bode P, Keller PM, et al. *Francisella tularensis* periprosthetic joint infections diagnosed with growth in cultures. J Clin Microbiol. 2019;57:e00339–19. 10.1128/JCM.00339-19 31189580 PMC6663894

[12] Rawal H, Patel A, Moran M. Unusual case of prosthetic joint infection caused by *Francisella tularensis.* BMJ Case Rep. 2017;2017:2017. 10.1136/bcr-2017-221258 PMC5652388 29025776

[13] Cooper CL, Van Caeseele P, Canvin J, Nicolle LE. Chronic prosthetic device infection with *Francisella tularensis.* Clin Infect Dis. 1999;29:1589–91. 10.1086/313550 10585830

[14] French Infectious Diseases Society (SPILF). Clinical recommendations for prosthetic joint infection by the Société de pathologie infectieuse de langue française [in French] [cited 2021 Jan 12]. https://www.infectiologie.com/fr/recommandations.html

[15] Beam E, Osmon D. Prosthetic joint infection update. Infect Dis Clin North Am. 2018;32:843–59. 10.1016/j.idc.2018.06.005 30241717

[16] Maurin M, Pelloux I, Brion JP, Del Banõ JN, Picard A. Human tularemia in France, 2006-2010. Clin Infect Dis. 2011;53:e133–41. 10.1093/cid/cir612 22002987

[17] World Health Organization. WHO guidelines on tularaemia. Geneva: The Organization: 2007 [cited 2020 Apr 7]. https://apps.who.int/iris/handle/10665/43793

[18] Kugeler KJ, Pappert R, Zhou Y, Petersen JM. Real-time PCR for *Francisella tularensis* types A and B. Emerg Infect Dis. 2006;12:1799–801. 10.3201/eid1211.060629 17283646 PMC3372352

[19] Caspar Y, Maurin M. *Francisella tularensis* susceptibility to antibiotics: a comprehensive review of the data obtained *In vitro* and in animal models. Front Cell Infect Microbiol. 2017;7:122. 10.3389/fcimb.2017.00122 28443249 PMC5386985

[20] Brunet CD, Hennebique A, Peyroux J, Pelloux I, Caspar Y, Maurin M. Presence of *Francisella tularensis* subsp. *holarctica* DNA in the Aquatic Environment in France. Microorganisms. 2021;9:1398. 10.3390/microorganisms9071398 34203503 PMC8306966

[21] Caspar Y, Hennebique A, Maurin M. Antibiotic susceptibility of *Francisella tularensis subsp. holarctica* strains isolated from tularaemia patients in France between 2006 and 2016. J Antimicrob Chemother. 2018;73:687–91. 10.1093/jac/dkx460 29253157

[22] Ferguson RJ, Palmer AJ, Taylor A, Porter ML, Malchau H, Glyn-Jones S. Hip replacement. Lancet. 2018;392:1662–71. 10.1016/S0140-6736(18)31777-X 30496081

[23] Price AJ, Alvand A, Troelsen A, Katz JN, Hooper G, Gray A, et al. Knee replacement. Lancet. 2018;392:1672–82. 10.1016/S0140-6736(18)32344-4 30496082

[24] Kapadia BH, Berg RA, Daley JA, Fritz J, Bhave A, Mont MA. Periprosthetic joint infection. Lancet. 2016;387:386–94. 10.1016/S0140-6736(14)61798-0 26135702

[25] Allegranzi B, Zayed B, Bischoff P, Kubilay NZ, de Jonge S, de Vries F, et al.; WHO Guidelines Development Group. New WHO recommendations on intraoperative and postoperative measures for surgical site infection prevention: an evidence-based global perspective. Lancet Infect Dis. 2016;16:e288–303. 10.1016/S1473-3099(16)30402-9 27816414

[26] Berríos-Torres SI, Umscheid CA, Bratzler DW, Leas B, Stone EC, Kelz RR, et al.; Healthcare Infection Control Practices Advisory Committee. Centers for Disease Control and Prevention guideline for the prevention of surgical site infection, 2017. JAMA Surg. 2017;152:784–91. 10.1001/jamasurg.2017.0904 28467526

[27] Tande AJ, Patel R. Prosthetic joint infection. Clin Microbiol Rev. 2014;27:302–45. 10.1128/CMR.00111-13 24696437 PMC3993098

[28] Zimmerli W, Trampuz A, Ochsner PE. Prosthetic-joint infections. N Engl J Med. 2004;351:1645–54. 10.1056/NEJMra040181 15483283

[29] Debarge R, Nicolle MC, Pinaroli A, Ait Si Selmi T, Neyret P. [Surgical site infection after total knee arthroplasty: a monocenter analysis of 923 first-intention implantations] [in French]. Rev Chir Orthop Repar Appar Mot. 2007;93:582–7. 10.1016/S0035-1040(07)92680-X 18065867

[30] Million M, Bellevegue L, Labussiere AS, Dekel M, Ferry T, Deroche P, et al. Culture-negative prosthetic joint arthritis related to *Coxiella burnetii.* Am J Med. 2014;127:786.e7–10. 10.1016/j.amjmed.2014.03.013 24662624

[31] van Hoek ML. Biofilms: an advancement in our understanding of *Francisella* species. Virulence. 2013;4:833–46. 10.4161/viru.27023 24225421 PMC3925715

[32] Centers for Disease Control and Prevention, American Society for Microbiology, Association of Public Health Laboratories. Basic protocols for Level A laboratories for the presumptive identification of *Francisella tularensis* [cited 2021 Jan 12]. https://www.epa.gov/sites/production/files/2015-07/documents/cdc-ftularemia.pdf

[33] Ellis J, Oyston PCF, Green M, Titball RW. Tularemia. Clin Microbiol Rev. 2002;15:631–46. 10.1128/CMR.15.4.631-646.2002 12364373 PMC126859

[34] Petersen JM, Schriefer ME, Gage KL, Montenieri JA, Carter LG, Stanley M, et al. Methods for enhanced culture recovery of *Francisella tularensis.* Appl Environ Microbiol. 2004;70:3733–5. 10.1128/AEM.70.6.3733-3735.2004 15184180 PMC427758

[35] Karatuna O, Celebi B, Can S, Akyar I, Kilic S. The use of Matrix-assisted laser desorption ionization-time of flight mass spectrometry in the identification of *Francisella tularensis.* Bosn J Basic Med Sci. 2016;16:132–8. 26773181 10.17305/bjbms.2016.894 PMC4852995

[36] López-Ramos I, Hernández M, Rodríguez-Lázaro D, Gutiérrez MP, Zarzosa P, Orduña A, et al. Quick identification and epidemiological characterization of *Francisella tularensis* by MALDI-TOF mass spectrometry. J Microbiol Methods. 2020;177:106055. 10.1016/j.mimet.2020.106055 32918935

[37] Regoui S, Hennebique A, Girard T, Boisset S, Caspar Y, Maurin M. Optimized MALDI TOF mass spectrometry identification of *Francisella tularensis* Subsp. holarctica. Microorganisms. 2020;8:1143. 10.3390/microorganisms8081143 32731606 PMC7464108

[38] Forsman M, Sandström G, Sjöstedt A. Analysis of 16S ribosomal DNA sequences of *Francisella* strains and utilization for determination of the phylogeny of the genus and for identification of strains by PCR. Int J Syst Bacteriol. 1994;44:38–46. 10.1099/00207713-44-1-38 8123561

[39] Versage JL, Severin DDM, Chu MC, Petersen JM. Development of a multitarget real-time TaqMan PCR assay for enhanced detection of *Francisella tularensis* in complex specimens. J Clin Microbiol. 2003;41:5492–9. 10.1128/JCM.41.12.5492-5499.2003 14662930 PMC309004

[40] Johansson A, Farlow J, Larsson P, Dukerich M, Chambers E, Byström M, et al. Worldwide genetic relationships among *Francisella tularensis* isolates determined by multiple-locus variable-number tandem repeat analysis. J Bacteriol. 2004;186:5808–18. 10.1128/JB.186.17.5808-5818.2004 15317786 PMC516809

[41] Osmon DR, Berbari EF, Berendt AR, Lew D, Zimmerli W, Steckelberg JM, et al.; Infectious Diseases Society of America. Diagnosis and management of prosthetic joint infection: clinical practice guidelines by the Infectious Diseases Society of America. Clin Infect Dis. 2013;56:e1–25. 10.1093/cid/cis803 23223583

[42] Tande AJ, Gomez-Urena EO, Berbari EF, Osmon DR. Management of prosthetic joint infection. Infect Dis Clin North Am. 2017;31:237–52. 10.1016/j.idc.2017.01.009 28366224

[43] Hepburn MJ, Simpson AJH. Tularemia: current diagnosis and treatment options. Expert Rev Anti Infect Ther. 2008;6:231–40. 10.1586/14787210.6.2.231 18380605

[44] Bossi P, Tegnell A, Baka A, van Loock F, Werner A, Hendriks J, et al. Bichat guidelines for the clinical management of tularaemia and bioterrorism-related tularaemia. Euro Surveill. 2004;9:27–8. 10.2807/esm.09.12.00503-en 29183485

[45] Thabit AK, Fatani DF, Bamakhrama MS, Barnawi OA, Basudan LO, Alhejaili SF. Antibiotic penetration into bone and joints: An updated review. Int J Infect Dis. 2019;81:128–36. 10.1016/j.ijid.2019.02.005 30772469

